# Ventriculitis due to multidrug-resistant gram-negative bacilli associated with external ventricular drain: evolution, treatment, and outcomes

**DOI:** 10.3389/fneur.2024.1384206

**Published:** 2024-04-26

**Authors:** Ana Luisa Corona-Nakamura, Martha Judith Arias-Merino, Eleazar Iván Ávila-Esparza, María de Lourdes Tolentino-Corona, César Cuauhtémoc Cañedo-Castañeda, Héctor Enrique Flores-Salinas, Juan Fernando Corona-Macías, Martha Elena Vázquez-Arias

**Affiliations:** ^1^Department of Internal Medicine, Mexican Institute of Social Security, Western National Medical Center, High Specialty Medical Unit, Guadalajara, Mexico; ^2^Research Committee, Western Clinical Research Institute, Zapopan, Mexico; ^3^Department of Neurosurgery, Mexican Institute of Social Security, Western National Medical Center, High Specialty Medical Unit, Guadalajara, Mexico; ^4^University Center for Health Sciences, University of Guadalajara, Guadalajara, Mexico

**Keywords:** external ventricular drain, EVD-associated ventriculitis, gram-negative bacilli, multidrug-resistant, mortality hazard ratios

## Abstract

**Introduction:**

Nosocomial infectious ventriculitis caused by multidrug-resistant (MDR) Gram-negative bacilli associated with external ventricular drainage (EVD) placement poses a significant mortality burden and hospital costs.

**Objectives:**

This study aims to analyze the characteristics, ventriculitis evolution, treatment, and outcomes of patients with ventriculitis due to MDR Gram-negative bacilli associated with EVD placement.

**Methods:**

A retrospective cohort study focusing on patients with nosocomial infection caused by MDR Gram-negative bacilli while on EVD was conducted from 2019 to 2022. Medical, laboratory, and microbiological records were collected. The antibiotic resistance of the Gram-negative bacilli isolated in the cerebrospinal fluid (CSF) of patients was analyzed. The risk factors were identified using univariate risk models and were analyzed using survival curves (Cox regression). An adjusted Cox proportional hazards model was also constructed.

**Results:**

Among 530 patients with suspected EVD-associated ventriculitis, 64 patients with isolation of Gram-negative bacilli in CSF were included. The estimated mortality was 78.12%. Hemorrhages (intracranial, subarachnoid, and intraventricular) were observed in 69.8% of patients. *Acinetobacter baumannii, Klebsiella pneumoniae*, and *Pseudomonas aeruginosa* were the most frequently isolated bacilli. In the univariate analysis, significant risk factors for mortality included arterial hypertension, a Glasgow Coma Scale (GCS) score of ≤ 8, invasive mechanical ventilation (IMV) upon hospital admission and during hospitalization, septic shock, and ineffective treatment. The adjusted Cox proportional hazards model revealed that septic shock (HR = 3.3, 95% CI = 1.5–7.2; *p* = 0.003) and ineffective treatment (HR = 3.2, 1.6–6.5, 0.001) were significant predictors. A high resistance to carbapenems was found for *A. baumannii* (91.3%) and *P. aeruginosa* (80.0%). Low resistance to colistin was found for *A. baumannii* (4.8%) and *P. aeruginosa* (12.5%).

**Conclusion:**

Ineffective treatment was an independent hazard factor for death in patients with ventriculitis caused by MDR Gram-negative bacilli associated with EVD.

## Introduction

Antimicrobial resistance is one of the significant threats to global public health. The World Health Organization estimated that antimicrobial resistance was directly attributable to 1.27 million deaths worldwide in 2019 (1). Among healthcare-associated postoperative infections, patients with neuroinfections experience prolonged hospital stays, along with high hospital costs, and are at a high risk of death (2–4). Among these neuroinfections, ventriculitis associated with external ventricular drain (EVD) placement has a reported prevalence between 0% and 22% (2, 5).

EVDs serve as a common temporary treatment in patients who have high intracranial pressure secondary to acute hydrocephalus caused by blockage of the cerebrospinal fluid (CSF) circulation. Generally, such blockage are caused by tumor obstruction, followed by chronic infectious meningitis, hemorrhage (intraventricular, subarachnoid, and intracranial), cranial trauma, CSF leak, and decompressive craniectomy (2, 6, 7).

Placement of a foreign body into a normally sterile (e.g., ventricular) cavity facilitates the entry of skin flora and pathogenic microorganisms present in the hospital environment. Patients who have undergone ventriculostomy are highly vulnerable because of their underlying condition and can acquire a nosocomial infection (frequently caused by multidrug-resistant (MDR) microorganisms). In addition, many antibiotics may not penetrate the ventricles (8).

Tunkel et al. (2) reported four mechanisms that occur most frequently in CSF shunts that enable a patient to acquire infections: (a) colonization of the shunt at the time of surgery, (b) retrograde infection from the distal end of the shunt, (c) through the skin while inserting a needle to collect CSF for culture or injecting a drug into the ventricular reservoir and (d) hematogenous seeding due to bacteremia. Sweid et al. (9) reported EVD replacement, CSF leak, and prolonged stay in the hospital to be risk factors associated with a higher risk of ventriculostomy infection.

New headaches, fever, nausea, vomiting, lethargy, and changes in mental status may be indicative of infectious ventriculitis. However, patients who have suffered a hemorrhage or who have a subarachnoid tumor may also present these clinical features (2).

Often, the diagnosis of ventriculitis is difficult to establish because it has an insidious onset and patients have unusual symptoms (10). The most important test to establish the diagnosis of ventriculitis is CSF culture. According to the Infectious Diseases Society of America (IDSA), several features are indicative of an infection associated with an EVD: single or multiple positive CSF cultures, pleocytosis and/or hypoglycorrhachia, and clinical symptoms suggesting ventriculitis or meningitis (2).

The most frequent bacterial pathogens isolated in the CSF of patients with ventriculitis associated with EVD use are skin commensals, such as coagulase-negative *Staphylococcus* species (including *Staphylococcus aureus*) and Gram-negative bacilli, such as *Acinetobacter baumannii, Pseudomonas aeruginosa, Klebsiella pneumoniae*, and *Escherichia coli* (3, 5). Solo-Peleteiro et al. (11) reported 75 bacterial isolates from CSF samples from patients with EVD-associated ventriculitis and found that nearly 65% of them corresponded to Gram-positive cocci and 28% to Gram-negative bacilli (most frequently *E. coli, A. baumannii*, and *K. pneumoniae*).

Studies of the problems associated with nosocomial infections (particularly infections caused by MDR Gram-negative bacilli) can clarify the risk factors of nosocomial infections, formulation of pathogen-surveillance protocols, and risk of death. The aim of this study was to enrich national/international information on these topics to provide useful data to improve daily clinical practice in hospitals.

## Materials and methods

A retrospective cohort study was designed covering the period 2019–2022. We included hospitalized patients who had undergone a neurosurgical procedure, who required an EVD, and who presented with nosocomial ventriculitis caused by MDR Gram-negative bacilli.

The present study was designed in two sections. In the first section, we analyzed data from patients selected according to the following case definition: Patients with CSF culture positive for Gram-negative bacilli (with isolation of a single type of bacteria) collected in the operation theater during replacement of an EVD in the first event of nosocomial infection.

“Nosocomial ventriculitis” was defined to originate after a neurosurgical procedure (placement/replacement of an EVD) in the hospital. Clinical suspicion of infection was based on IDSA criteria: (1) having a positive CSF culture (for Gram-negative bacilli isolates); (2) having at least two of the following symptoms: new or worsening altered mental status (the presence of a new headache, nausea, lethargy, and/or altered state of consciousness); and fever (>38.0°C) in the absence of another focus of infection. The cytochemical analysis of CSF was done to identify hypoglycorrhachia, hyperproteinorrachia, and pleocytosis. Magnetic resonance imaging and computed tomography of the cranium were used only to make a differential diagnosis (these data were not included in the present study) (2, 12).

The inclusion criteria were patients aged ≥17 years having nosocomial ventriculitis after neurosurgery caused by EVD replacement by Neurosurgery and Infectious Diseases Services. Positivity of CSF culture found after EVD replacement was considered (IDSA criteria). The exclusion criteria were as follows: patients with post-neurosurgical ventriculitis due to placement/replacement of an internal ventricular drain (ventriculoperitoneal shunt valve) and those with incomplete medical records (2). Data for the study were collected from medical, laboratory, and microbiological records.

Several variables were studied, including the primary disease that necessitated ventriculostomy as well as conditions upon hospital admission (hereafter termed “admission”) and during hospitalization: neurological status (based on the Glasgow Coma Scale (GCS) score; invasive mechanical ventilation (IMV); septic shock; number of ventriculostomies; EVD duration (days); hospitalization duration; evolution of ventriculitis; treatment; outcomes; and laboratory and microbiological findings). Patients were divided into two groups: “survivors” and “non-survivors.”

The mortality prognostic factors of patients with ventriculitis selected based on isolated Gram-negative bacilli were also analyzed. Upon admission, these prognostic factors included a low GCS score (3–8), a history of arterial hypertension, and IMV. During hospitalization, these prognostic factors included the number of ventriculostomies before infection, presentation of septic shock, resistance to carbapenems, and receiving ineffective treatment (non-efficacious treatment). The latter understood was in relation to the presence of MDR Gram-negative bacilli (with resistance to ≥3 families of antibiotics), particularly resistance to carbapenems, and patients who received a suboptimal dose of polymyxin E (colistin; <14 days) (13, 14).

In the second section, a complementary analysis of the Gram-negative bacilli isolated from the CSF samples was done. Positive cultures taken through the EVD outside theater were excluded. We also excluded CSF samples considered to be contaminated or that had been colonized by pathogens. Positive CSF cultures from patients without symptoms of infection or pleocytosis and/or hypoglycorrhachia were considered to be contaminated. Colonization was considered if multiple CSF cultures or positive Gram stains were found with a normal CSF cell count without clinical symptoms suggesting ventriculitis or if normal concentrations of glucose and protein in CSF were observed (2).

Parameters set by the Clinical and Laboratory Standards Institute (CLSI) and the European Committee on Antimicrobial Susceptibility Testing (EUCAST, 2020) were used to interpret antibiotic susceptibility. The epidemiological cutoff values (minimum inhibitory concentration of wild-type organisms) were ≥2 mg/L for the most important members of *Enterobacteriaceae* and *Acinetobacter* species and ≥4 mg/L for *P. aeruginosa* (15).

### Statistical analyses

In the first section, a description of the demographic variables, primary disease, and clinical status of patients upon admission and during hospitalization, ventriculitis evolution, treatment, and outcomes was made. The microbiological and cytochemical findings of CSF were also documented. The frequency distribution of data was analyzed by the Kolmogorov–Smirnov test. Median values and interquartile ranges (IQRs) were used if the data did not have a normal distribution. The receiver operating characteristics (ROC) curves were created to establish points between survivors and non-survivors in terms of quantitative variables. Using a univariate model, a risk analysis was carried out between survivors and non-survivors. Significant mortality risk factors were analyzed using survival curves (Cox regression) considering admission as the initial time and patient death as the final event. The effects of the set of variables suggesting a death poor prognosis on the hazard ratio (HR) were analyzed through the construction of an adjusted Cox proportional hazards model (*p* < 0.05). An additional univariate analysis was undertaken to analyze the factors suggesting a poor prognosis for patients with ventriculitis based on isolated Gram-negative bacilli.

In the second section, resistance to different antibiotics by bacterial isolates in CSF was described. The resistance found according to isolated Gram-negative bacilli, as well as risk estimation by univariate analysis, was also described.

Univariate analyses were done using the Mann–Whitney *U*-test. Qualitative variables were examined using the χ^2^ test or Fisher's exact test. The results were considered significant if the *p* < 0.05. SPSS 25 (IBM, Armonk, NY, USA) was employed for statistical analyses.

## Results

**Figure 1:**
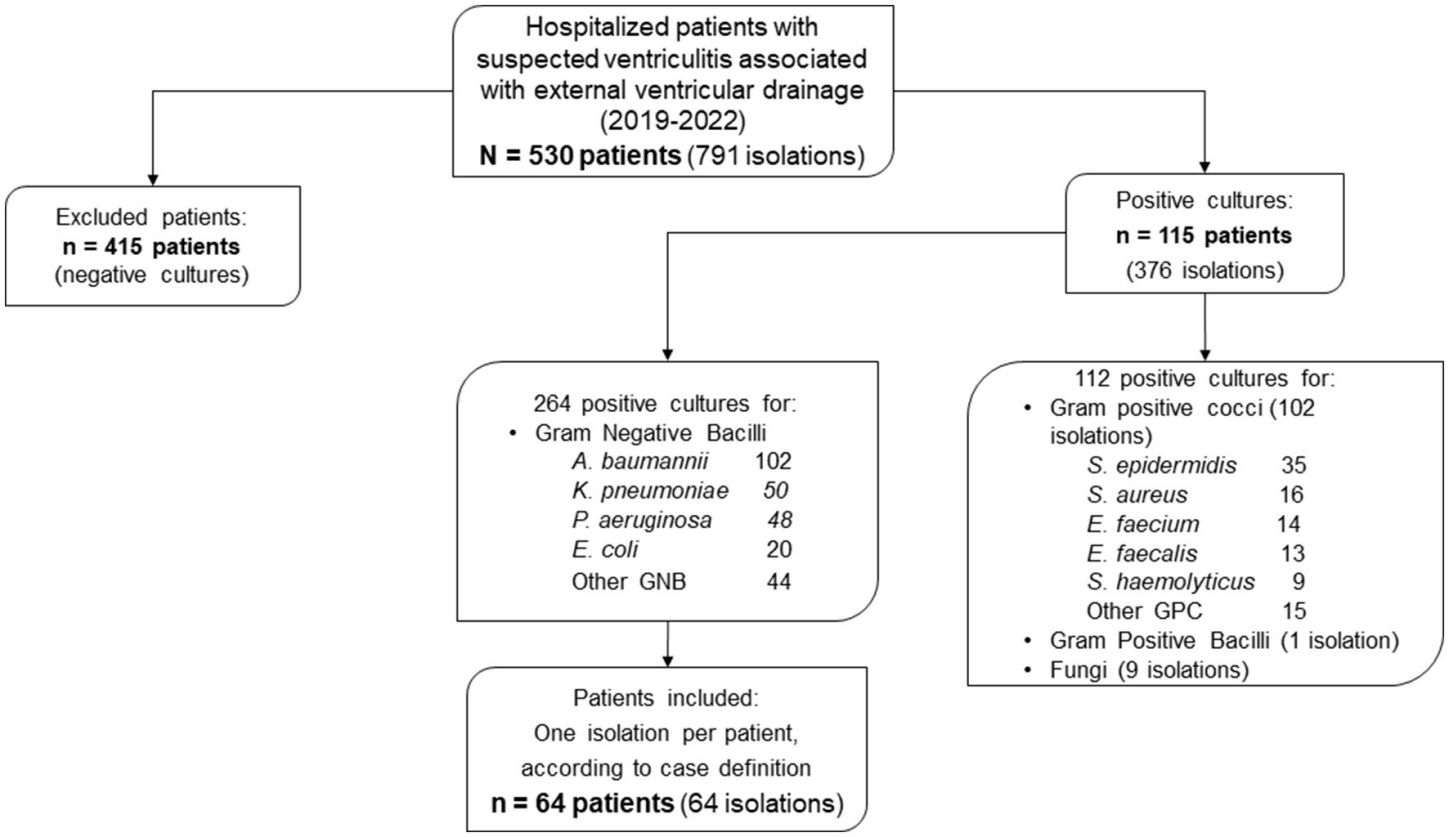
Participant flowchart.

Of 530 patients with suspected ventriculitis associated with an EVD between 2019 and 2022, 791 isolates from CSF samples were obtained. A total of 415 patients (78.3%) with negative cultures were excluded. Of the remaining patients, 115 (21.7%) had 376 positive cultures. A total of 112 isolates were eliminated from patients with positive cultures for Gram-positive cocci or bacilli and fungi and 102 (91.1%) from patients with positive cultures for Gram-positive cocci. Of the 264 samples in which Gram-negative bacilli were found, *A. baumannii* was found in 102 (38.6%). According to the case definition, 64 patients were included in our analysis (Figure 1).

All EVDs were replaced in theater. In all cases, the type of catheter replaced was not impregnated with antibiotics or silver nanoparticles.

The median age of patients was 51 (range, 17–82) years, and 33 were men (51.6%). The group of non-survivors was 50/64 patients (78.12%).

Hemorrhagic brain events (intracranial, subarachnoid, or intraventricular hemorrhage; 69.8%), decompressive craniectomy (31.7%) aneurysm (22.2%), CSF fistula (20.6%), ischemic stroke (16.1%), obstructive brain tumor (14.3%), traumatic brain injury (12.9%), arteriovenous malformation (12.7%), brain herniation (3.2%), and tubercular meningitis (1.6%) were the primary diseases suffered by the patients.

Upon admission, 52 patients (81.3%) had hydrocephalus. Arterial hypertension was found in 29 out of 63 (46.0%) of cases. Forty-six patients (73%) were found to have neurological deficits. Twenty-four patients (39.3%) had a GCS score of 3–8. Twenty-six patients (44.8%) required IMV upon admission. During hospitalization, 47 patients required IMV (78.3%) and 31 patients (49.2%) presented with septic shock. The duration of hospitalization was 3–169 (median, 30; IQR, 14–50) days. A median of 10 (IQR, 7–16) days for each ventriculostomy per patient was estimated (Table 1). All CSF samples had pleocytosis, with a median of 695 (IQR, 131–2173) cells/μL and 66.7% had ≥200 cells/μL. In addition, 57.1% of samples had hypoglycorrhachia (<0.30 g/L), and hyperproteinorrachia (≥1 g/L) was noted in 87.3% of samples.

**Table 1:** Demographic characteristics, comorbidities, and complications of patients with ventriculitis due to MDR Gram-negative bacilli.

**Variables**	**Total, *N =* 64 (%)**	**Non-survivors *n* = 50 (%)**	**Survivors *n =* 14 (%)**	**OR (CI)**	***p*-value**
Male patients	33 (51.6)	27 (54.0)	6 (42.9)		0.332
Age (years), median, IQR	51 (43–62)	51 (40–60)	60 (48–63)		0.148
**Primary disease**					
Cerebral hemorrhagic events	44/63 (69.8)	37/49 (75.5)	7 (50.0)		0.069
Decompressive craniectomy	20/63 (31.7)	16/49 (32.7)	4 (29.6)		1.000
Aneurysm	14/63 (22.2)	13/49 (26.5)	1 (7.1)		0.162
CSF fistula	13/63 (20.6)	9/49 (18.4)	4 (28.6)		0.461
Ischemic CVE	10/62 (16.1)	7/48 (14.6)	3 (21.4)		0.681
Obstructive brain tumor	9/63 (14.3)	5/49 (10.2)	4 (28.6)		0.101
Traumatic brain injury	8/62 (12.9)	8/48 (16.7)	0		NS
Arteriovenous malformation	8/63 (12.7)	6/49 (12.2)	2 (14.3)		1.000
Brain herniation	2/63 (3.2)	2/48 (4.1)	0		NS
Tuberculous meningitis	1/64 (1.6)	1/50 (2.0)	0		NS
**Conditions upon admission and during hospitalization**					
Hydrocephalus	52 (81.3)	42 (84.0)	10 (71.4)		0.438
Arterial hypertension	29/63 (46.0)	27/49 (55.1)	2 (14.3)	7.4 (1.5–36.4)	0.013
Neurological deficit upon admission	46/63 (73.0)	38/49 (77.6)	8 (57.1)		0.121
GCS score upon admission, median, IQR	15 (10–18)	9 (6–14)	14 (10–15)		0.026
GCS score 3- 8 upon admission	24/61 (39.3)	23/47 (48.9)	1 (7.1)	12.5 (1.5–103.1)	0.005
IMV upon admission	26/58 (44.8)	24/44 (54.5)	2 (14.3)	7.2 (1.4–36.0)	0.012
IMV during hospitalization	47/60 (78.3)	40/46 (87.0)	7 (50.0)	6.7 (1.7–25.8)	0.007
Septic shock	31/63 (49.2)	30/49 (61.2)	1/14 (7.1)	20.5 (2.5–169)	0.000
Hospitalization (days), median, IQR, range	30 (14–50), (3–169)	29 (14–47), (3–44)	37 (17–60), (9–169)		0.385
Number of ventriculostomies, median, IQR	2 (1–3)	2 (1–3)	1 (1–3)		0.293
Days ventriculostomies, median, IQR	10 (7–16)	10 (7–13)	12 (7–21)		0.293

CI, Confidence interval; IQR, Interquartile range; CVE, Cerebral Vascular Event; GCS, Glasgow Coma Scale; IMV, Invasive mechanical ventilation

In CSF cultures, 26 (40.6%) were positive for *A. baumannii*, 11 (17.2%) for *K. pneumoniae*, 10 (15.6%) for *P. aeruginosa*, 8 (12.5%) for *E. coli*, and the remaining 9 (14.1%) for other Gram-negative bacilli (*Enterobacter cloacae, Stenotrophomonas maltophilia, Proteus mirabilis*, and *Pseudomonas stutzeri*).

We discovered that 32 out of 58 samples (55.2%) showed resistance to carbapenems, 31 out of 57 (54.4%) to meropenem, 56 out of 61 (91.8%) to third-generation cephalosporins, and 45 out of 60 (75.0%) to cefepime. Resistance to quinolones was noted in 46 out of 58 (79.3%) of samples, 37 out of 58 (63.8%) to aminoglycosides, 24 out of 55 (43.6%) to tigecycline, and 5 out of 47 (10.6%) to colistin (Table 2).

**Table 2:** Cytochemical and microbiological findings in CSF and treatment of patients with ventriculitis due to MDR Gram-negative bacilli.

**Variable**	**Total, *N =* 64 (%)**	**Non-survivors n=50 (%)**	**Survivors n=14 %)**	**OR (CI)**	***p*-value**
**Laboratory and microbiological findings in CSF**					
Leukocytes in CSF (cells/μL), IQR	695 (131–2173)	690 (135–2680)	700 (77–1790)		0.786
CSF leukocytes ≥200 cells/μL	38/57 (66.7)	30/44 (68.2)	8 (61.5)		0.742
CSF glucose <0.30 g/L	32/56 (57.1)	24/43 (55.8)	8 (61.5)		0.760
CSF proteins ≥1g/L	48/55 (87.3)	37/42 (88.1)	11 (84.6)		0.664
*A. baumannii*	26 (40.6)	23 (46.0)	3 (21.4)		0.129
*K. pneumoniae*	11 (17.2)	7 (14.0)	4 (28.6)		0.237
*P. aeruginosa*	10 (15.6)	7 (14.0)	3 (21.4)		0.677
*E. coli*	8 (12.5)	5 (10.0)	3 (21.4)		0.357
Other Gram-Negative Bacilli	9 (14.1)	8 (16.0)	1 (7.1)		0.670
Resistance to carbapenem	32/58 (55.2)	29/46 (63.0)	3/12 (25.0)	5.1 (1.2–21.5)	0.025
Resistance to meropenem	31/57 (54.4)	28/45 (62.2)	3/12 (25.0)	4.9 (1.2–20.8)	0.027
Resistance to imipenem	28/48 (58.3)	25/40 (62.5)	3/8 (37.5)		0.251
Resistance to third-generation cephalosporins	56/61 (91.8)	44/48 (91.7)	12/13 (92.3)		1.000
Resistance to cefepime	45/60 (75.0)	35/47 (74.5)	10/13 (76.9)		1.000
Resistance to quinolones	46/58 (79.3)	38/45 (84.4)	8/13 (61.5)		0.116
Resistance to aminoglycosides	37/58 (63.8)	28/45 (62.2)	9/13 (69.2)		0.751
Resistance to tigecycline	24/55 (43.6)	19/43 (44.2)	5/12 (41.7)		1.000
Resistance to colistin resistance ¥	5/47 (10.6)	3/38 (7.9)	2/9 (22.2)		0.240
**Treatment**					
Carbapenem resistance, IV colistin	15/30 (50.0)	12/27 (44.4)	3/3 (100.0)		NS
Carbapenem resistance, IV + IVT colistin	4/30 (13.3)	3/27 (11.1)	1/3 (33.3)		0.360
Ineffective treatment according to resistance	26/63 (41.3)	24/49 (49.0)	2/14 (14.3)	5.8 (1.2–28.5)	0.030

CI, Confidence interval; IQR, Interquartile range; CSF, Cerebrospinal fluid; Other Gram-Negative Bacilli: E. cloacae, S. maltophilia, P. mirabilis and P. stutzeri. ¥ Cut-off level for colistin resistance ≥2mg/L, except for P. aeruginosa (≥4 mg/L) (EUCAST, 15). IV, Intravenous; IVT, Intraventricular.

In the univariate analysis, several risk factors were associated with death: having arterial hypertension [odds ratio (OR) = 7.4, 95% confidence interval (CI) = 1.5–36.4, *p* = 0.013]; a GCS score of 3–8 (12.5, 1.5–103, 0.005); IMV upon admission (7.2, 1.4–36.0, 0.012); IMV during hospitalization (6.7, 1.7–25.8, 0.007); septic shock (20.5, 2.5–169, 0.000) (Table 1).

In relation to the univariate analysis of the resistance of Gram-negative bacilli to antibiotics, resistance to carbapenems (5.1, 1.2–21.5, 0.025) and meropenem (4.9, 1.2–20.8, 0.027) was significant. Receiving ineffective treatment (non-effective treatment) in relation to antibiotic resistance was also a risk factor associated with death (5.8, 1.2–28.5, 0.030) (Table 2).

**Figure 2:**
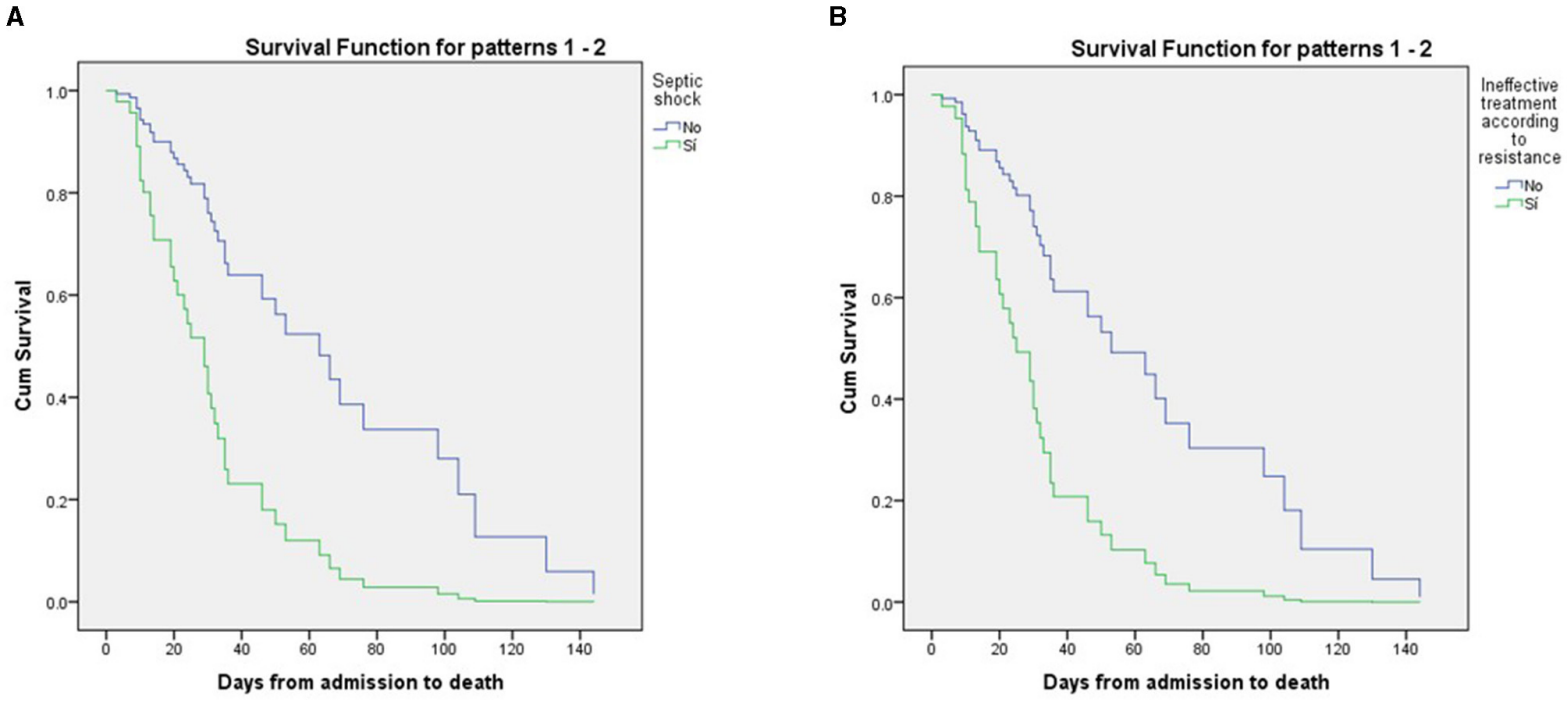
Patients survival analysis using cox regression **(A)** Septic shock and **(B)** ineffective treatment.

To construct the adjusted Cox model, several prognostic variables that were significant in the univariate analysis were taken into consideration: arterial hypertension, low GCS score upon admission, IMV upon admission and during hospitalization, septic shock, and ineffective treatment. Table 3 shows the results of the adjusted Cox proportional hazards model. Septic shock (HR = 3.3, CI 1.5–7.2, *p* = 0.003) and ineffective treatment (HR = 3.2, CI 1.6–6.5, *p* = 0.001) were significant. The patient survival graphs of these two hazard conditions for mortality are shown in Figure 2.

**Table 3:** Adjusted Cox proportional hazards model.

**Variable**	**HR (95% CI)**	***p*-value**
Arterial hypertension	1.5 (0.7–3.1)	0.292
GCS score 3- 8 upon admission	1.4 (0.7–2.7)	0.320
Septic shock	3.3 (1.5–7.2)	0.003
Ineffective treatment	3.2 (1.6–6.5)	0.001

HR, Hazard Ratio; CI, Confidence interval; GCS, Glasgow Coma Scale.

Table 4 shows the factors leading to a poor prognosis according to Gram-negative bacilli most frequently observed in isolates from the CSF cultures of patients, and the main bacteria were *A. baumannii, P. aeruginosa, K. pneumoniae*, and *E. coli*. Carbapenem resistance was reported in 21 out of 23 (91.3%) of *A. baumannii* isolates and 23 out of 26 (88.5%) of *A. baumannii* isolates were from non-survivors. For patients with *A. baumannii* isolates, the requirement for IMV upon admission (OR = 4.9, CI = 1.6–15.3, *p* = 0.006), as well as the resistance to carbapenems for *A. baumannii* (OR = 22.9, CI = 4.6–115.4, *p* = 0.000) were estimated to be risk factors.

**Table 4:** Poor prognostic factors in patients with ventriculitis due to external ventricular drain (2019–2022).

	***Acinetobacter baumannii***			***Pseudomonas aeruginosa***		
	***n** =* **26 (%)**	**OR (CI)**	**p**	***n** =* **10 (%)**	**OR (CI)**	***p*** **-value**
Arterial hypertension	13 (50.0)		0.392	6 (60.0)		0.492
GCS score upon admission 3- 8	13/25 (52.0)		0.078	3 (30.0)		0.726
IMV upon admission	15/22 (68.2)	4.9 (1.6–15.3)	0.006	4 (40.0)		1.000
Carbapenem resistance	21/23 (91.3)	22.9 (4.6–115.4)	0.000	8 (80.8)		0.160
Septic shock	13 (50.0)		0.560	5 (50.0)		1.000
Ventriculostomies ≥4	7 (26.9)		0.219	2 (20.0)		1.000
Ineffective treatment	13 (50.0)		0.179	4 (40.0)		1.000
Non-survivors	23 (88.5)		0.129	7 (70.0)		0.677
	***Klebsiella pneumoniae***			***Escherichia coli***		
	***n** =* **11 (%)**	**OR (CI)**	**p**	***n** =* **8 (%)**	**OR (CI)**	***p*** **-value**
Arterial hypertension	6 (54.5)		0.741	2/7 (28.6)		0.437
GCS score upon admission 3- 8	3 (27.3)		0.502	2/7 (28.6)		0.694
IMV upon admission	2 (18.2)		0.090	3/7 (42.9)		1.000
Carbapenem resistance	1 (9.1)		NS	0		NS
Septic shock	4/10 (40.0)		0.732	5 (62.5)		0.474
Ventriculostomies ≥4	2 (18.2)		1.000	0		NS
Ineffective treatment	4 (36.4)		1.000	2/7 (28.6)		0.690
Non-survivors	7 (63.6)		0.237	5 (62.5)		0.357

GCS, Glasgow Coma Scale; CI, Confidence interval; IMV, Invasive mechanical ventilation.

### Gram-negative bacilli isolated

The following resistances of the selected isolates to different antibiotics were found: 91.8% for cephalosporins, ceftriaxone (84.6%), ceftazidime (80.0%), cefepime (75.0%); 79.3% for ciprofloxacin; 74.0% for ampicillin/sulbactam; 73.3% for aztreonam; 53.3% for gentamicin; and 55.2% for carbapenems (imipenem 58.3%, meropenem 54.4%). The highest sensitivities were obtained for colistin (resistance of 10.6%) followed by amikacin (resistance of 40%) (Table 5).

**Table 5:** Resistance to antibiotics of all isolated bacilli in patients with ventriculitis (2019–2022).

**Antimicrobial**	**2019**	**2020**	**2021**	**2022**	**Total**
	*n =* 10 (%)	*n =* 13 (%)	*n* = 23 (%)	*n =* 15 (%)	*N =* 61 (%)
Carbapenems	2/7 (28.6)	8 (61.5)	14 (60.9)	8 (53.3)	32/58 (55.2)
Ertapenem	0	0	1/9 (11.1)	1/7 (14.3)	2/24 (8.3)
Imipenem	-	8 (61.5)	12/20 (60.0)	8 (53.3)	28/48 (58.3)
Meropenem	1/6 (16.7)	8 (61.5)	14 (60.9)	8 (53.3)	31/57 (54.4)
Piperacillin/tazobactam	1/5 (20.0)	11 (84.6)	11/20 (55.0)	7/14 (50.0)	30/52 (57.7)
Ampicillin/sulbactam	6 (60.0)	9/11 (81.8)	12/17 (70.6)	10/12 (83.3)	37/50 (74.0)
Cephalosporins	8 (80.0)	13 (100.0)	20 (87.0)	15 (100.0)	56 (91.8)
Cefoxitin	-	2/3 (66.7)	2/9 (22.2)	3/6 (50.0)	7/18 (38.9)
Ceftazidime	-	11 (84.6)	17 (73.9)	12/14 (85.7)	40/50 (80.0)
Ceftriaxone	8 (80.0)	11/12 (91.7)	13/17 (76.5)	12/13 (92.3)	44/52 (84.6)
Cefepime	8 (80.0)	10 (76.9)	16 (69.6)	11/14 (78.6)	45/60 (75.0)
Aminoglycosides	6 (60.0)	10 (76.9)	13 (56.5)	8/14 (57.1)	37/60 (61.7)
Amikacin	3/7 (42.9)	2/6 (33.3)	9/18 (50.0)	2/9 (22.2)	16/40 (40.0)
Gentamicin	4 (40.0)	10 (76.9)	11 (47.8)	7/14 (50.0)	32/60 (53.3)
Ciprofloxacin	6 (60.0)	10/12 (83.3)	19/22 (86.4)	11/14 (78.6)	46/58 (79.3)
Tigecycline	3/9 (33.3)	6/13 (46.2)	8/21 (38.1)	7/12 (58.3)	24/55 (43.6)
Colistin^*^	-	1 (7.7)	2/21 (9.5)	2/13 (15.4)	5/47 (10.6)
Aztreonam	7/9 (77.8)	-	2/3 (66.7)	2/3 (66.7)	11/15 (73.3)

* Cutoff for colistin resistance ≥2mg/L, except for P. aeruginosa (≥4 mg/L) (15).

Table 6 shows the antimicrobial resistance described for each isolated Gram-negative bacillus. For *A. baumannii*, resistance was found to piperacillin/tazobactam, third-generation cephalosporins and amikacin (100%), ciprofloxacin (96.0%), cefepime (92.3%), carbapenems (91.3%) (meropenem 90.9% and imipenem 90.5%), gentamicin (73.1%), tigecycline (52.0%), and colistin (4.8%). For *P. aeruginosa*, resistance was found to third-generation cephalosporins (100%), carbapenems (80.0%), piperacillin/tazobactam (71.4%), amikacin (66.7%), cefepime (55.6%), and colistin (12.5%). For *K. pneumoniae*, resistance was found to third-generation cephalosporins (72.7%), ciprofloxacin (70.0%), aminoglycosides (54.5%), extended-spectrum β-lactamase (50.0%), and colistin (25.0%). For *E. coli*, resistance was found to third-generation cephalosporins (87.5%), ciprofloxacin (62.5%), aminoglycosides (50.0%), gentamicin (50.0%), and extended-spectrum β-lactamases (100%).

**Table 6:** Antimicrobial resistance in patients with ventriculitis (2019–2022).

	***Acinetobacter baumannii***			***Pseudomonas aeruginosa***		
**Antimicrobial**	***n** =* **26 (%)**	**OR (IC)**	***p*** **-value**	***n** =* **10 (%)**		***p*** **-value**
Carbapenems	21/23 (91.3)	22.9 (4.6–115.4)	0.000	8/10 (80.0)		0.160
Imipenem	19/21 (90.5)	19.0 (3.6–100.2)	0.000	7/8 (87.5)		0.116
Meropenem	20/22 (90.9)	21.8 (4.3–110.2)	0.000	8/10 (80.0)		0.092
Piperacillin/tazobactam	21/21 (100.0)		NA	5/7 (71.4)		0.685
Ampicillin/sulbactam	21/26 (80.8)		0.339	2/2 (100.0)		NA
Third-generation cephalosporins	26/26 (100.0)		NA	10/10 (100.0)		NA
Ceftazidime	22/22 (100.0)		NA	5/9 (55.6)		0.065
Cefepime	24/26 (92.3)	7.4 (1.5–36.8)	0.008	5/9 (55.6)		0.208
Aminoglycosides	19/26 (73.1)		0.093	6/9 (66.7)		1.000
Amikacin	6/6 (100.0)		NA	6/9 (66.7)		0.120
Gentamicin	19/26 (73.1)	4.4 (1.4–13.3)	0.007	4/9 (44.4)		0.721
Ciprofloxacin	24/25 (96.0)	12.0 (1.4–100.1)	0.008	1/5 (20.0)		1.000
Tigecycline	13/25 (52.0)		0.193	NA		NA
Colistin	1/21 (4.8)		0.365	1/8 (12.5)		1.000
	***Klebsiella pneumoniae***			***Escherichia coli***		
**Antimicrobial**	***n** =* **11 (%)**	**OR (IC)**	***p*** **-value**	***n** =* **8 (%)**	**OR (IC)**	***p*** **-value**
Carbapenems	1/11 (9.1)		NS	0		NA
Ertapenem	1/11 (9.1)		1.000	0		NA
Imipenem	1/8 (12.5)		NS	0		NA
Meropenem	1/11 (9.1)		NS	0		NA
Piperacillin/tazobactam	2/11 (18.2)		NS	1/8 (12.5)		NS
Ampicillin/sulbactam	7/11 (63.6)		0.445	6/8 (75.0)		1.000
Third-generation cephalosporins	8/11 (72.7)		NS	7/8 (87.5)		0.518
Ceftazidime	5/8 (62.5)		0.331	5/7 (85.7)		1.000
Ceftriaxone	7/11 (63.6)		0.051	6/7 (85.7)		1.000
Cefepime	7/11 (63.6)		0.442	7/8 (87.5)		0.666
Aminoglycosides	6/11 (54.5)		0.504	4/8 (50.0)		0.443
Amikacin	2/11 (18.2)		0.148	0		NA
Gentamicin	5/11 (45.5)		0.740	4/8 (50.0)		1.000
Ciprofloxacin	7/10 (70.0)		0.417	5/8 (62.5)		0.342
Tigecycline	1/9 (11.1)		0.062	0		NA
Colistin¶	2/8 (25.0)		0.196	1/7 (14.3)		0.571
Aztreonam	2/3 (66.7)		1.000	3/4 (75.0)		1.000
TMP-SMX	2/3 (66.7)		1.000	1/1 (100.0)		NA
ESBL	5/10 (50.0)		0.058	6/6 (100.0)		NA

CI, Confidence interval; TMP-SMX, Trimethoprim/sulfamethoxazole; ESBL, Extended-spectrum β-lactamase; NS, Not significant; NA, Not applicable. ^¶^Cutoff for colistin resistance ≥2mg/L, except for P. aeruginosa (≥4 mg/L) (15).

In the univariate analysis, the risk factors for resistance were observed only in *A. baumannii* isolates. To carbapenems with OR 22.9 (4.6–115.4), *p* = 0.000 (including imipenem and meropenem); cefepime with OR 7.4 (1.5–36.8), *p* = 0.008; gentamicin with OR 4.4 (1.4–13.3), *p* = 0.007, and ciprofloxacin with OR 12.0 (1.4–100.1), *p* = 0.008.

## Discussion

According to the case definition, 64 patients who met the eligibility criteria were selected. Ventriculitis is considered to be an unusual complication, which is potentially fatal. The prognosis is worse if ventriculitis is associated with an infection by MDR Gram-negative bacilli (particularly *A. baumannii*) (10, 16).

The most serious problem in nosocomial infections associated with neurosurgery is ventriculitis, which has a poor prognosis and causes death or the development of a vegetative state in up to 70% of patients. Ventriculitis becomes even more complex in patients who acquire *A. baumannii* infection, with nosocomial meningitis due to this bacillus causing death in up to 72.7% of cases (8, 16, 17). In our study, there was a high mortality, estimated at 78.12% (50 of 64 patients died). Of the 26 patients with *A. baumannii* in CSF, 23 (88.5%) died.

We registered 530 patients with suspected ventriculitis associated with an EVD. Of these, 115 (21.7%) patients developed microbiological ventriculitis associated with an EVD, which was slightly lower than that reported by Dorresteijn et al. (18) (23.0%) in a meta-analysis. In addition, the main indication for EVD replacement was identical: hemorrhage (cerebral, intracranial, subarachnoid, intraventricular) estimated at 69.8% in our study and 69.0% in the study of Dorresteijn et al. (18).

The prevalence of EVD-associated infections has been reported to range from 0.0 to 23.2%. In a retrospective cohort study in Austria between 2008 and 2019, out of 396 patients, 32 patients (8.1%) with EVD-associated infections were reported. CSF samples mainly contained Gram-positive cocci, and only three patients (9.3%) had Gram-negative bacilli in their samples (19).

There are epidemiological differences in the prevalence of causative agents between countries. A higher prevalence of Gram-negative bacilli has been reported in some developing countries. However, a growing trend in the prevalence of these bacilli is observed globally, mainly *P. aeruginosa, A. baumannii*, and *K. pneumoniae* resistant to carbapenems (12, 16).

Most of our patients had hydrocephalus. An EVD is frequently used for the management of acute hydrocephalus. Hydrocephalus can arise as a secondary complication of hemorrhage (intraventricular or subarachnoid) or as a complication following an inflammatory reaction (infectious or non-infectious) (20, 21).

A significant number of patients presented with neurological deficits upon admission and required IMV. Ventriculitis is difficult to diagnose clinically because it is masked by the disease that led to EVD placement (22).

If cytochemical parameters in CSF are inconclusive, the diagnosis of ventriculitis is difficult. One must determine if the test results are due to inflammation after neurosurgery, device placement, or infection. In our study, a positive CSF culture for Gram-negative bacilli was an inclusion criterion. Hence, the diagnosis was confirmed in all patients according to IDSA 2017 criteria. Most patients had pleocytosis (≥200 cells/μL), hypoglycorrhachia, and hyperproteinorrachia. After EVD insertion, pleocytosis (mean count ≥175 cells/μL) in CSF is a decisive indicator of infection (2).

The importance of *A. baumannii* as an MDR pathogen found at a high frequency in patients with ventriculitis associated with EVD use has been highlighted in several studies (6, 23, 24). Herein, the prevalence of *A. baumannii* in CSF was estimated to be 40.6%. It was the highest in relation to other Gram-negative bacilli isolated from CSF (followed by *K. pneumoniae* at 17.2%). Ye et al. (23) reported, in a retrospective study of patients with ventriculitis/meningitis, a prevalence of 39.1% of *A. baumannii* in CSF samples.

In our univariate analysis, arterial hypertension, a low GCS score (≤8), IMV upon admission and during hospitalization, the presentation of septic shock, resistance to carbapenems, and receiving ineffective treatment were risk factors for death. Sharma et al. (25) undertook a study on the post-neurosurgical infection (meningitis/ventriculitis) by *Acinetobacter* species. In their univariate analysis, arterial hypertension, a low GCS score, and septic shock were risk factors for death. They also reported, using a multivariate Cox proportional hazards model, age ≥40 years, pleocytosis (≥200 cells/μL), and having several comorbidities to be risk factors for death. Panic et al. (26) used logistic regression analysis for patients with *A. baumannii* infection. They found IMV and GCS scores ≤ 8 to be associated with a poor outcome (26). In our adjusted analysis of the Cox proportional hazards model, the presentation of septic shock and ineffective treatment resulted in significant HRs.

A favorable prognosis for patients with nosocomial ventriculitis depends on the speed with which treatment is initiated. Hospital environments with MDR Gram-negative bacilli have been associated with an increased risk of death from meningitis/ventriculitis, isolation of *A. baumannii*, and administration of inappropriate empiric treatments (12, 27).

A significant number of our non-survivor patients had *A. baumannii* in CSF samples. The next most prevalent pathogens found in CSF were *P. aeruginosa, K. pneumoniae*, and *E. coli*. The risks of a poor prognosis were associated with *A. baumannii* isolates in CSF samples that required IMV upon admission and if *A. baumannii* was also resistant to carbapenems.

### Gram-negative bacilli isolated

For the strains of Gram-negative bacilli isolated in CSF samples, clear resistance to cephalosporins, ciprofloxacin, ampicillin/sulbactam, and aztreonam was observed. Our data on antibiotic resistance were similar to those reported by Ye et al. (23). However, their data indicated higher antibiotic resistance rates compared to our results for ceftazidime (80.0% vs. 85.9%), ceftriaxone (84.6% vs. 93.9%), cefepime (75.0% vs. 82.6%), ciprofloxacin (79.3% vs. 80.2%), ampicillin/sulbactam (74.0% vs. 79.7%), aztreonam (73.3% vs. 76.5%), gentamicin (53.3% vs. 71.8%), and amikacin (40.0% vs. 53.2%). In relation to carbapenems, the resistance rate estimated by Ye et al. (23) was much higher than that in the bacterial strains isolated in our study (e.g., imipenem, 58.3% vs. 97.8%; meropenem, 54.4% vs. 81.9%). Resistance to colistin was low in both studies (10.6% vs. 11.6%).

The *A. baumannii* isolates in CSF samples exhibited high resistance (73.1%−100%) to piperacillin/tazobactam, amikacin, third- and fourth-generation cephalosporins, carbapenems (meropenem and imipenem), and gentamicin. Similar results were found for *P. aeruginosa* isolates in CSF samples. Low resistance to colistin was found in both bacilli. Isolates of *K. pneumoniae* and *E. coli* did not show significant resistance to carbapenems.

For *A. baumannii* isolates, an increased risk of resistance to carbapenems, cefepime, gentamicin, and ciprofloxacin was found. Panic et al. (26) reported *A. baumannii* to be the most prevalent bacillus in CSF samples from patients in a hospital specializing in infectious diseases, and they found resistance to carbapenems in all samples.

According to the IDSA 2017 guidelines, in patients with nosocomial ventriculitis/meningitis due to Gram-negative bacilli, empirical management is with meropenem. Colistin should be only administered if there is resistance to carbapenems (2). Several authors have reported the high resistance of Gram-negative bacilli to carbapenems (especially *A. baumannii*, >40%), so the recommended empirical treatment should be concomitant use of colistin intravenous (IV) and intraventricular (IVT) (4, 14, 24).

Given the minimal resistance to colistin found by our bacterial isolates, this indication would be appropriate for our patients. However, in cases of an inadequate response to systemic antimicrobial therapy using colistin (IVT and IV), colistin combined with another drug should be attempted. Tigecycline (IVT) combined with colistin (IVT) could be a good option against carbapenem-resistant *A. baumannii* and *K. pneumoniae*. However, in the case of our *A. baumannii* isolates in CSF samples, tigecycline resulted in high resistance (52.0%) (28–30).

Treatment with colistin (IVT) has been recommended to ensure penetration of this antibiotic into ventricles, but it should be carried out simultaneously with IV administration. The dose and time of administration must be specified (i.e., strict monitoring of treatment must be maintained) (2, 4, 24).

A co-formulation of ceftazidime/avibactam (IV) could be a good treatment option for neurosurgical interventions against *P. aeruginosa* and *Enterobacteriaceae* (*K. pneumoniae and E. coli*) that produce KPC-type carbapenemases (31).

Ineffective treatment was an independent factor that affected mortality directly. The therapeutic challenge is very great for infections of the central nervous system. One must deepen knowledge of the pharmacokinetics and pharmacodynamics of antibiotics and establish better recommendations for their use. Randomized clinical trials and prospective cohort studies are needed to accurately determine the potential benefit of treatments targeting MDR pathogens (32, 33).

### Limitations

The retrospective design of our study implied limitations. There was high variability regarding the prevalence of EVD-associated infectious ventriculitis and mortality. These variations (in addition to the clinical particularities of patients) were due to the different definitions of infectious ventriculitis, patient selection criteria, and the methodology used in different studies. Our study cohort was collected during the coronavirus disease-2019 pandemic, as well as conjunctural changes in the México Health System that directly influenced patient care in our hospital.

## Conclusions

In the placement/replacement of an EVD, in addition to the disease severity of the patients who require it, multiple factors must be considered: maintaining strict standards of healthcare inside and outside theaters; adequate availability of and accessibility to antibiotics; and synchronization with the microbiological laboratory. Thus, providing patients with a timely diagnosis and efficacious treatment that ensures more favorable outcomes, reduced stay in hospital, low healthcare costs, and low risk of death is crucial. Despite the similarities identified in previous studies with our patients and our microbiological data, each hospital center must face the problems of its own environment as well as the clinical particularities of its target population. Our hospital usually receives patients with diseases in advanced stages of illness and from all over Western Mexico, hence the complexity of our hospital care.

## Data availability statement

The raw data supporting the conclusions of this article will be made available by the authors, without undue reservation.

## Ethics statement

The studies involving humans were approved by the Institutional Review Board of the Western National Medical Center of the Mexican Institute of Social Security with Registration numbers: R-2021–1301–221 and R-2023–1301–036. The studies were conducted in accordance with the local legislation and institutional requirements. The participants provided their written informed consent to participate in this study.

## Author contributions

AC-N: Writing – review & editing, Writing – original draft. MA-M: Writing – review & editing, Writing – original draft. EÁ-E: Writing – review & editing. MT-C: Writing – review & editing. CC-C: Writing – review & editing. HF-S: Writing – review & editing. JC-M: Writing – review & editing. MV-A: Writing – review & editing.
